# Comparison of Treatment Safety and Patient Survival in Elderly versus Nonelderly Patients with Advanced Hepatocellular Carcinoma Receiving Sorafenib Combined with Transarterial Chemoembolization: A Propensity Score Matching Study

**DOI:** 10.1371/journal.pone.0117168

**Published:** 2015-02-17

**Authors:** Hao Hu, Zhenhua Duan, Xiaoran Long, Yancu Hertzanu, Xiaoqiang Tong, Xiaoquan Xu, Haibin Shi, Sheng Liu, Zhengqiang Yang

**Affiliations:** 1 Department of Interventional Radiology, The First Affiliated Hospital of Nanjing Medical University, Nanjing, China; 2 Chengdu Center for Disease Control and Prevention, Chengdu, China; 3 School of Pharmacy, Anhui Medical University, Hefei, China; 4 Faculty of Health Sciences, Ben-Gurion University of the Negev, Beer-Sheva, Israel; 5 Department of Interventional Radiology and Vascular Surgery, Peking University First Hospital, Beijing, China; 6 Department of Radiology, The First Affiliated Hospital of Nanjing Medical University, Nanjing, China

## Abstract

**Aims:**

This retrospective study was carried out to compare the outcomes between elderly (≥70 years of age) and nonelderly patients (<70 years of age) with advanced hepatocellular carcinoma (HCC) who received sorafenib combined with transarterial chemoembolization (TACE).

**Methods:**

88 patients with a confirmed diagnosis of advanced HCC were enrolled in this study. Of these, 24 elderly patients were matched with 48 nonelderly patients at a 1:2 ratio using propensity score matching to minimize selection bias. The related adverse events and survival benefits were compared between the two groups.

**Results:**

Sorafenib combined with TACE was equally well tolerated in both age groups, and grade 3 or 4 adverse events were similarly observed in 54.2% of elderly and 50.0% of nonelderly patients (P = 0.739). There were no significant differences in survival time between the elderly and nonelderly patients (P = 0.876). Significant prognostic factors for overall survival as identified by multivariate analysis were the Child–Pugh score and portal vein invasion.

**Conclusions:**

Sorafenib combined with TACE may be well tolerated and effective in elderly patients with advanced HCC. Age alone is not a parameter for the treatment of advanced HCC patients.

## Introduction

Hepatocellular carcinoma (HCC) is a common cancer with an increasing incidence [1], especially in the elderly population (defined as ≥70 years old in the present study) [2, 3]. Medical comorbidities, impaired organ function, altered drug pharmacokinetics, poor functional status, and symptoms caused by additional tumors [4] are often seen in elderly patients with cancer, making them less tolerant of and/or less amenable to various systemic treatments [3, 5, 6]. Elderly patients have always been deemed no longer fit in the past for palliative treatments, such as transarterial chemoembolization (TACE), percutaneous ethanol injection (PEI), radiofrequency ablation (RFA), and oral sorafenib. However, recent studies have shown that when appropriate patient selection criteria are adopted, age does not influence treatment efficacy in elderly patients with HCC [3, 7].

Transarterial chemoembolization (TACE) is an effective palliative treatment in patients with middle- and advanced-stage HCC. Old age was previously considered to be a relative contraindication to TACE in the management of HCC [8, 9]; however, recent studies describing different experiences with TACE have reported equivalent outcomes between young and old patients [3, 10–12]. Several studies on sorafenib treatment in elderly patients demonstrated that the increasing age does not affect the tolerance of sorafenib [13, 14]. However, all of these studies only considered the clinical efficacy of a single treatment in elderly patients.

Even with the development of pharmaceutical and interventional techniques, a single treatment currently has limited clinical efficacy for this poorly controllable disease. New treatment strategies are urgently required [15, 16]. Combined therapy, such as sorafenib combined with TACE, has displayed unique advantages in the management of HCC [17, 18]. Sorafenib, which is an orally administered small molecule, inhibits multiple protein kinases. At present, sorafenib is the only approved systemic therapy for patients with advanced stage (BCLC-C) [19], and phase III randomized clinical trials demonstrate that it is efficacious for prolonging time-to-progression (TTP) and median survival of patients with HCC [20,21]. TACE is one of the most commonly used treatments for unresectable HCC. Current guidelines recommend TACE as the standard treatment of BCLC-B patients [22]. Recently, a growing body of research shows that TACE is effective for controlling symptoms of patients with advanced HCC, including those with vascular invasion or metastases, and is a common mainstay palliative modality in Asia [23,24]. However, the effects of advancing age on clinical outcomes and tolerance to this combined therapy are largely unknown.

In this study, we compared the tolerance to and efficacy of sorafenib combined with TACE between elderly (≥70 years old) and nonelderly patients (<70 years old) with advanced HCC.

## Materials and Methods

### Patients

We retrospectively analyzed a consecutive cohort of advanced HCC patients, with vascular invasion and/or distant metastasis corresponding to Barcelona Clinic Liver Cancer (BCLC) stage C, who were admitted to our department from March 2009 to November 2013. This retrospective comparative study was approved by the ethics committee of the first affiliated hospital of Nanjing medical university. Written informed consent was obtained from each patient before treatment. All patients’ medical records were accessed using their real names, which were anonymized before analysis. After the pretreatment investigations, our multidisciplinary treatment team of radiologists, hepatologists, and oncologists selected suitable patients to receive sorafenib combined with TACE. The diagnosis of HCC was reconfirmed in all patients by histological or cytological examination or on the basis of the American Association for the Study of Liver Diseases practice guidelines [25].

The inclusion criteria were advanced HCC (the presence of Barcelona Clinic Liver Cancer (BCLC) stage C disease involving vascular invasion and/or extrahepatic spread), an Eastern Cooperative Oncology Group performance status of 0 to 2, and Child–Pugh class A or B liver disease. The exclusion criteria were as follows: a history of liver transplantation; only nodal or distant metastases without viable liver lesions; any secondary malignancies; a history of concomitant use of another targeting agent, chemotherapy, or immunotherapy; and loss to follow-up. The TACE procedure and sorafenib treatment have previously described [26].

### Follow-up

All TACE and sorafenib-related adverse events (AEs) were graded according to the National Cancer Institute Common Toxicity Criteria Adverse Events version 3.0. Laboratory tests performed during follow-up included hematologic and biochemical evaluations (e.g., complete blood cell count), prothrombin time measurement, and liver function evaluation. To assess liver function, the aspartate aminotransferase, alanine aminotransferase, alpha-fetoprotein, bilirubin, and albumin levels were measured every 4 weeks. Dynamic liver computed tomography or magnetic resonance imaging was performed every 6 to 8 weeks to evaluate the treatment response. If necessary, chest computed tomography and/or a bone scans were also performed to diagnose extrahepatic metastasis.

### Statistical analysis

A 1:2 propensity score-matched analysis was performed to adjust for potential bias. This method is frequently used in observational studies because it allows for nonrandomized group assignments. We used nearest-neighbor matching with no replacement (a single participant could not be selected multiple times) to match patients in the nonelderly group with those in the elderly group (Stata command psmatch2; StataCorp LP, College Station, TX, USA) [27].

Differences between the two age groups were evaluated with Fisher’s exact test or the chi-squared test for categorical variables and Student’s t-test or the Wilcoxon–Mann–Whitney test for continuous variables. We compared overall survival (OS) between the two propensity score-matched cohorts using the Kaplan–Meier method and log-rank test. Multivariable Cox proportional hazards models were used to determine the effect of age on OS after adjusting for the prognostic variables, and hazard ratios with 95% confidence intervals (CIs) were calculated.

All analyses were two-sided and performed at the 5% significance level. Statistical analyses were performed with SPSS software package version 13.0 (SPSS, Inc., Chicago, IL, USA) and Stata 12.0 (StataCorp LP).

## Results

### Patient characteristics

88 patients with advanced HCC were admitted to our department from March 2009 to November 2013 and received sorafenib combined with TACE. Of these patients, 64 (72.7%) belonged to the nonelderly group (<70 years old) and 24 (27.3%) belonged to the elderly group (≥70 years old). An Eastern Cooperative Oncology Group (ECOG) performance status of 0 (P = 0.021), prior procedures (P = 0.043), and nodular HCC (P = 0.042) were more frequent in the nonelderly group. There were no between-group differences in liver cirrhosis, etiology, Child–Pugh score, ascites, extrahepatic tumor metastasis or portal vein invasion, or serum alpha-fetoprotein levels.

After performing nearest-neighbor matching (1:2) and based on the number of elderly patients, 48 nonelderly patients were matched for the analyses. The characteristics of the patients are listed in Table 1. No significant differences in the baseline characteristics of the patients were observed between the two groups after propensity score matching. In total, 24 elderly patients (33.3%; mean age, 76 years; range, 70–83 years) and 48 nonelderly patients (66.7%; mean age, 57 years; range, 31–69 years) were included in this analysis. Most patients were male (83.8% and 79.2% in the nonelderly and elderly groups, respectively), consistent with the local epidemiology. The majority of patients in both groups had reasonable liver function (Child–Pugh class A liver disease) prior to treatment; 68.8% of patients in the nonelderly group and 70.8% of patients in the elderly group had Child–Pugh class A liver disease (P = 0.856). Most patients in both age groups had an ECOG performance status score of 0 to 1 (68.7% and 58.3% in the nonelderly and elderly groups, respectively), although more elderly patients than nonelderly patients had a performance status score of 2 (31.3% and 41.7% in the nonelderly and elderly groups, respectively). The median follow-up period for all patients was 9.6 months (range, 1.8–39.7 weeks).

**Table 1:** Baseline characteristics of each age group before and after propensity score matching.

Characteristics	Parameter	Age≥70 Years (N = 24)	Age <70 Years (pre-match, N = 64)	P value	Age <70 Year (pre-match, N = 48)	P value
Gender	Male	19 (79.2%)	56 (87.5%)	0.330	40 (83.3%)	0.665
	Female	5 (20.8%)	8 (12.5%)		8 (16.7%)	
Age (years)	Mean±SD	75.54±4.64	56.38±8.97	<0.001	57.21±9.28	<0.001
	Range	70–83	31–69		31–69	
ECOG Performance Status	0	1 (4.2%)	20 (31.3%)	0.014	8 (16.7%)	0.285
	1	13 (54.2%)	29 (45.3%)		25 (52.1%)	
	2	10 (41.7%)	15 (23.4%)		15 (31.3%)	
Prior procedures	Yes	15 (62.5%)	53 (82.8%)	0.043	37 (77.1%)	0.864
	No	9 (37.5%)	11 (17.2%)		11 (22.9%)	
Cirrhosis	Yes	14 (58.3%)	42 (65.6%)	0.621	30 (62.5%)	0.732
Etiology	Hepatitis B	13 (54.2%)	41 (64.1%)	0.464	30 (62.5%)	0.755
	Hepatitis C	3 (12.5%)	5 (7.8%)	0.678	4 (8.3%)	0.574
	No infection	8 (33.3%)	18 (28.1%)	0.793	14 (29.2%)	0.717
Child-Pugh score	A	17 (70.8%)	44 (68.8%)	0.850	33 (68.8%)	0.856
	B	7 (29.2%)	20 (31.3%)		15 (31.3%)	
Type of tumor	Nodular	10 (41.7%)	42 (65.6%)	0.042	26 (54.2%)	0.317
	Infiltrative	14 (58.3%)	22 (43.4%)		22 (45.8%)	
Tumor metastasis	Main portal vein invasion	4 (16.7%)	12 (18.8%)	0.821	9 (18.8%)	0.828
	Portal vein branch invasion	8 (33.3%)	17 (26.6%)	0.530	14 (29.2%)	0.717
	Distant tumor metastasis	8 (33.3%)	25 (39.1%)	0.621	15 (13.1%)	0.717
	Both	4 (16.7%)	10 (15.6%)	0.905	10 (20.8%)	0.828
Ascites	Yes	18 (75.0%)	46 (71.9%)	0.821	39 (81.2%)	0.538
Alfa-fetoprotein (ng/mL)	>400 ng/mL	13 (54.2%)	42 (65.6%)	0.323	29 (60.4%)	0.612
Total bilirubin umol/L	Mean±SD	27.18±9.82	27.45±9.06	0.434	28.44±8.67	0.503
	>34 umol/L	7 (29.2%)	14 (21.9%)	0.475	11 (22.9%)	0.564
Albumin	Mean±SD	37.18±5.99	37.53±6.18	0.416	36.95±6.28	0.485
	<35 g/L	10 (41.7%)	24 (37.5%)	0.721	21 (43.8%)	0.866
INR	Mean±SD	1.06±0.19	1.09±0.21	0.878	1.09±0.23	0.854
	>1.7	0 (0%)	1 (1.6%)	0.538	1 (2.1%)	0.476
Number of treatments (TACE)	1	10 (41.7%)	19 (29.7%)	0.171	18 (37.5%)	0.732
	2	8 (33.3%)	26 (40.6%)	0.532	18 (37.5%)	0.729
	3	4 (16.7%)	12 (18.8%)	0.821	9 (18.8%)	0.828
	>3	2 (8.3%)	7 (10.9%)	0.720	3 (6.3%)	0.743

SD, standard deviation; ECOG, Eastern Cooperative Oncology Group; INR, international normalized ratio; TACE, transarterial chemoembolization

### Safety and tolerability

Sorafenib combined with TACE was well tolerated in all patients in both age groups (Table 2). TACE-related grade 3 or 4 abdominal pain was more common in elderly (4.2%) than in nonelderly patients (2.1%), and the incidence of grade 3 or 4 nausea and/or vomiting was lower in elderly (0.0%) than in nonelderly patients (4.2%). However, neither difference was statistically significant (P = 0.612 and 0.310, respectively). The most common sorafenib-induced AEs of all grades were hand-foot skin reaction, diarrhea, fatigue, alopecia, hypertension, and thrombocytopenia. The observed proportions of patients with sorafenib-related grade 3 or 4 hypertension and thrombocytopenia were larger in elderly patients (12.5% and 4.2%, respectively) than in nonelderly patients (4.2% and 0.0%, respectively), and the incidence of grade 3 or 4 hand-foot skin reaction, diarrhea, and alopecia were lower in elderly patients (8.3%, 8.3%, and 0.0%, respectively) than in nonelderly patients (10.4%, 10.4%, and 4.2%, respectively). Again, these differences were not statistically significant (P = 0.778, 0.778, and 0.310, respectively). Additionally, the proportions of other AEs did not differ significantly with age. Severe AEs, including gastrointestinal bleeding, hepatic encephalopathy, and hyperbilirubinemia, were observed in 4.2%, 8.3%, and 0.0% of the elderly patients, respectively. Dosage interruption due to AEs was observed in 45.8% and 33.3% of elderly and nonelderly patients, respectively, with no significant difference (P = 0.302).

**Table 2:** Comparison of combination therapy-related adverse events by age group.

Adverse events	Age≥70 years (N = 24)		Age<70 years (N = 48)		P value	
	All Grade	Grade 3 or 4	All Grade	Grade 3 or 4	All Grade	Grade 3 or 4
Any grade 3 or 4 toxicity	-	13 (54.2%)	-	24 (50.0%)	-	0.739
TACE-related symptom						
Fever	13 (54.2%)	0 (0%)	21 (43.8%)	0 (0%)	0.459	na
Abdominal pain	18 (75.0%)	1 (4.2%)	34 (70.8%)	1 (2.1%)	0.786	0.612
Nausea and/or vomiting	11 (45.8%)	0 (0%)	20 (41.7%)	2 (4.2%)	0.803	0.310
Iodine allergy and preparation	0 (0%)	0 (0%)	2 (4.2%)	1 (2.1%)	0.549	0.476
Sorafenib-related symptom						
HFSR	10 (41.7%)	2 (8.3%)	17 (35.4%)	5 (10.4%)	0.616	0.778
Diarrhea	14 (58.3%)	2 (8.3%)	25 (52.1%)	5 (10.4%)	0.802	0.778
Fatigue	3 (12.5%)	1 (4.2%)	10 (20.8%)	2 (4.2%)	0.522	0.710
Alopecia	5 (20.8%)	0 (0%)	7 (14.6%)	2 (4.2%)	0.518	0.310
Hypertension	7 (29.2%)	3 (12.5%)	11 (22.9%)	2 (4.2%)	0.576	0.190
Thrombocytopenia	9 (37.5%)	1 (4.2%)	25 (52.1%)	0 (0%)	0.318	0.333
Severe adverse event						
Gastrointestinal bleeding	4 (16.7%)	1 (4.2%)	6 (12.5%)	3 (6.3%)	0.722	0.716
Hepatic encephalopathy	3 (12.5%)	2 (8.3%)	2 (4.2%)	1 (2.1%)	0.325	0.211
Hyperbilirubinmia	6 (25.0%)	0 (0%)	9 (18.8%)	0 (0%)	0.552	na

na, not applicable; TACE, transarterial chemoembolization; HFSR, hand-foot skin reaction

### Overall survival

**Fig 1:**
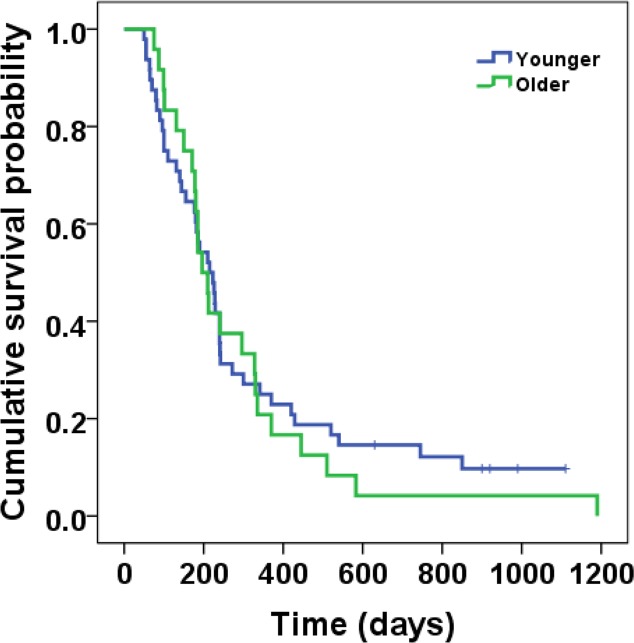
Kaplan–Meier analysis of overall survival between the two age groups.

Kaplan–Meier survival analysis indicated no significant difference in the median OS following combination therapy between elderly and nonelderly patients (6.5 [95% CI, 5.5–7.6] vs. 8.4 [95% CI, 5.6–8.8] months, respectively; P = 0.876) (Table 3 and Fig. 1). We performed subgroup survival analysis according to independent prognostic factors for OS identified in the multivariate analysis (Table 4). Age did not have a significant influence on OS after adjusting for other related risk factors (P = 0.507 for OS).

**Table 3:** Comparison of survival by age group.

Characteristics	Parameter	Age≥70 Years (n = 24)			Age<70 Years (n = 48)		
		N	mOS (95% CI) †	p-value	N	mOS (95% CI)	p-value
All patients		24	6.5 (5.5–7.6)	na	48	8.4 (5.6–8.8)	na
ECOG Performance Status	0	1	na	0.011	8	6.2 (1.7–10.7)	0.083
	1	13	10.9 (6.1–15.7)		25	7.2 (5.4–9.0)	
	2	10	5.7 (2.0–9.7)		15		7.6 (2.2–13.0)	
Child-Pugh class	A	18	7.1 (1.1–13.0)	0.011	33	7.5 (6.4–8.7)	0.128
	B	6	3.4 (0.1–6.6)		15	4.7 (1.4–7.9)	
Cirrhosis	No	10	6.2 (4.8–7.5)	0.716	18	7.6 (7.4–7.8)	0.300
	Yes	14	6.5 (4.6–8.5)		30	6.0 (4.4–7.6)	
Type of tumor	Nodular	10	7.0 (4.1–9.9)	0.413	26	6.2 (5.0–7.4)	0.538
	Infiltrative	14	6.2 (5.2–7.1)		22	7.6 (7.2–7.0)	
Tumor metastasis	MPVT/PVBT	12	7.0 (0.7–13.3)	0.026	23	6.0 (4.2–7.8)	0.591
	TEM	8	6.5 (5.0–8.1)		15	8.0 (5.6–10.4)	
	Both	4	2.9 (2.1–3.7)		10	7.6 (7.2–8.0)	
Ascites	No	6	10.9 (4.6–17.3)	0.073	9	7.0 (4.7–9.3)	0.511
	Yes	18	6.2 (5.8–6.5)		39	7.4 (5.5–8.4)	
Alfa-fetoprotein	≤400 ng/mL	11	7.0 (1.6–12.4)	0.12	19	6.3 (3.8–8.9)	0.943
	>400 ng/mL	13	6.2 (4.8–7.5)		29	7.6 (6.8–8.4)	
Total bilirubin	≤34 umol/L	17	6.5 (5.4–7.7)	0.497	37	7.5 (6.8–8.3)	0.035
	>34 umol/L	7	7.0 (1.3–6.9)		11	4.7 (0.8–8.6)	
INR	≤1.7	24	6.5 (5.5–7.6)	na	47	7.4 (5.6–9.3)	0.695
	>1.7	0	na		1	Na	

†Median survival calculated by Kaplan–Meier analysis

mOS, median overall survival; 95% CI, 95% confidence interval; na, not applicable; ECOG, Eastern Cooperative Oncology Group; INR, international normalized ratio; MPVT, main portal vein thrombosis; PVBT, portal vein branch thrombosis

**Table 4:** Cox proportional hazards multivariate regression analysis of overall survival.

Characteristics	Hazard ratio(95% CI)	P value
Age≥70 years	1.215 (0.683,2.162)	0.507
ECOG Performance Status score 1–2	0.973 (0.321,2.950)	0.961
Child-Pugh class B	0.817 (1.072,3.080)	0.027
Cirrhosis (positive)	0.900 (0.496,1.634)	0.730
Type of tumor (infiltrative)	0.973 (0.535,1.771)	0.929
Tumor metastasis (PVT without TEM)	0.297 (0.124,0.711)	0.006
Ascites (positive)	2.221 (0.783,6.301)	0.134
Alfa-fetoprotein>400ng/mL	0.604 (0.307,1.189)	0.144

ECOG, Eastern Cooperative Oncology Group; PVT, portal vein thrombosis; TEM, tumor extrahepatic metastasis

## Discussion

The optimal management of elderly patients with advanced HCC is an important issue because both the average age of patients and the incidence of HCC are increasing [28, 29]. Limited information on the efficacy and safety of combination treatment in elderly patients with advanced HCC is currently available. The present study has addressed this issue. The OS was similar in the elderly and nonelderly patient groups. Furthermore, there was no significant evidence that age affected survival after adjustment for other prognostic factors. Although more AEs were observed in elderly patients, these events were largely manageable, and the incidence of AEs was similar in both age groups.

We performed a case-control study of TACE combined with sorafenib versus TACE monotherapy to evaluate the treatment efficacy and safety in 246 patients with HCC [26]. Both univariate and multivariate analysis showed that age was not independently associated with OS. However, we did not compare safety and tolerance between the elderly and nonelderly patients. Therefore, in the present study, we comprehensively and thoroughly evaluated OS and AEs between elderly and nonelderly patients. Both the safety and tolerability in the present study population were similar to our previous experience with sorafenib and TACE.

Recent data were published concerning the safety and efficacy of sorafenib in a cohort of elderly patients from Hong Kong. In this population of >70-year-old patients with advanced HCC, the safety of sorafenib was shown to be equal to that in younger patients (grade 3 or 4 AEs, 68.6% and 62.7%, respectively; P = 0.56) [14]. More recently, the first non-Asian study [30] to investigate the use of sorafenib in a large cohort of elderly patients demonstrated that nonelderly patients who received sorafenib treatment were more susceptible to developing severe AEs than were older patients despite a lower rate of comorbidities (grade 3 or 4 AEs, 15.7% and 9.2%, respectively; P = 0.146). These results are similar to those obtained in another single-center study in Italy [31]. On the other hand, despite the poorer outcomes in elderly patients in a previous retrospective cohort study [32], a large case-control study recently performed by Yau et al. [33] and a 20-year multicenter experience from Italy indicated comparable efficacy of and tolerance to TACE for the treatment of advanced HCC in both young and elderly patients. More recently, TACE was reported to be safe and effective in selected very elderly patients with HCC [34]. However, more data are needed to confirm the safety of TACE in this population.

With respect to efficacy, recent studies have demonstrated similar median survival, regardless of age, following monotherapy in elderly (≤70 years old) and nonelderly patients (<70 years old). Wong et al. [14] compared the efficacy of sorafenib in elderly (n = 37) and nonelderly patients (n = 135) with advanced HCC. The OS was similar in the elderly and nonelderly groups (5.32 vs. 5.16 months, respectively; P = 0.310). They concluded that the survival benefits of sorafenib are comparable in elderly and nonelderly patients with advanced HCC. Likewise, these results are similar to those obtained in another single-center study in Italy (16 and 12 months in elderly and nonelderly group, respectively) [31]. Yau et al. [33] conducted a large comparative study of 1040 patients with HCC treated with TACE (197 elderly and 843 nonelderly). They concluded that TACE had comparable efficacy in elderly and nonelderly patients with HCC. Finally, a prospective cohort study conducted by Cohen et al. [34] also confirmed that advanced age was not associated with a decreased survival rate.

In an attempt to compensate for the low cure rate obtained with TACE and the resulting hypoxia-induced angiogenic activity, combination treatment with sorafenib is currently being evaluated for unresectable HCC in two phase-II trials. Both of these studies have shown promising results with tolerable toxicity profiles [17, 18]. Likewise, several continuous retrospective studies have further confirmed that combination therapy provides significantly better outcomes than does monotherapy for patients with advanced HCC [35, 36]. To the best of our knowledge, however, the safety and efficacy of sorafenib combined with TACE have not been previously compared. Based on the present results, combination therapy appears to be equally effective for elderly and nonelderly patients with advanced HCC. Recent studies have identified tumor stage, tumor markers, and hepatic functional reserve as prognostic factors that affect the survival of patients with HCC [33, 34, 37]. Similar to the reported results, the prognosis after combination therapy largely depends on the pretreatment liver function and tumor burden, but patient age is not a significant factor. An important management strategy for combination therapy is to ensure that no overlap occurs between the two treatments. To protect liver function in patients with HCC, oral sorafenib should not be administered within a 4- to 7-day window before or after the performance of TACE.

There were several important findings in this study. First, sorafenib combined with TACE was shown to be safe in elderly patients with advanced HCC. Second, the survival benefits of combination therapy were comparable in elderly and nonelderly patients with advanced HCC. Finally, the outcomes of this retrospective cohort study provide new insights and guidance for treating elderly patients with advanced HCC.

There are limitations to the present study. First, this was a single-center experience, and the results may not be generalizable to patients with HCC in other countries. Second, it was a retrospective study involving a relatively limited number of elderly patients, potentially leading to patient selection bias. Propensity score matching was used to mitigate the potential confounding selection bias of this nonrandomized trial. Third, with respect to the timing of sorafenib administration, we applied an interrupted approach that involved placing patients on sorafenib between TACE procedures and temporarily stopping treatment around the TACE procedure. This method may be insufficient to inhibit the surge of angiogenic factors seen shortly after TACE [38]. However, it most likely provides the best balance between safety and efficacy. Finally, despite the results of the current study demonstrate the efficacy and safety of combined therapy in elderly patients with a high functioning status and satisfactory medical condition; thus, our results cannot be extrapolated to the general elderly population with advanced HCC.

In conclusion, for patient with advanced HCC who conform to the inclusion criteria and do not have concomitant disease that would hinder therapy, sorafenib combined with TACE may be well tolerated and effective in elderly patients with advanced HCC. Further prospective randomized trials are needed to confirm the potential safety and benefit of sorafenib combined with TACE in elderly patients.
